# Myristic Acid Serum Levels and Their Significance for Diagnosis of Systemic Inflammatory Response, Sepsis, and Bacteraemia

**DOI:** 10.3390/jpm11040306

**Published:** 2021-04-16

**Authors:** Roman Zazula, Michal Moravec, František Pehal, Tomáš Nejtek, Marek Protuš, Martin Müller

**Affiliations:** 1 Department of Anesthesiology and Intensive Care, First Faculty of Medicine, Charles University and Thomayer University Hospital, 140 59 Prague, Czech Republic; michal.moravec@ftn.cz (M.M.); peh@post.cz (F.P.); tomas.nejtek@ftn.cz (T.N.); martin.muller@ftn.cz (M.M.); 2 Department of Anaesthesiology, Resuscitation and Intensive Care, Institute for Clinical and Experimental Medicine, First Faculty of Medicine, Charles University, 140 21 Prague, Czech Republic; marek.protus@ikem.cz

**Keywords:** myristic acid, SIRS, sepsis, bacteraemia, septic shock, gas chromatography/mass spectrometry (GC/MS), biomarker

## Abstract

Myristic acid is identified as a metabolite with the highest diagnostic sensitivity and specificity in the metabolome of patients with bacteraemia. Its significant decrease has been observed in patients with septic shock not responding to treatment. Another study has reported a close correlation of myristic acid levels with the outcome of severe trauma patients. Myristic acid concentrations were investigated in a cohort of septic patients and patients with Systemic Inflammatory Response Syndrome (SIRS) in 5 consecutive days following diagnosis and compared to healthy controls. The study population groups—Sepsis 34, SIRS 31, and Healthy Control 120 patients were included. Serum samples were analyzed using gas chromatography and mass spectrometry. The myristic acid levels in the Sepsis Group and SIRS Group were found to be significantly higher when compared to healthy controls. The serum concentration of myristic acid in septic patients with bacteraemia was higher than in septic patients without bacteraemia. Most patients with sepsis and SIRS had the highest levels of myristic acid within 24 h after an established diagnosis. Myristic acid should be considered as a new candidate marker of severe inflammation and sepsis. A simplified analysis and sufficient body of validated data are necessary steps towards the introduction of this metabolite into routine clinical practice.

## 1. Introduction

The last two decades have been marked by significant developments in the field of the so-called ‘precision medicine’, including the introduction of this concept in sepsis diagnosis and management. Using ‘omics’ (genomics, proteomics, and metabolomics) has contributed to the diagnosis of sepsis and helped in its prognostication and successful treatment [1,2,3]. An emerging subspeciality in the laboratory work-up, metabolomics, is becoming a powerful tool for clinical management of various diseases and syndromes. It captures, in the real clinical situation and time, the substrates and products of metabolism which are influenced by both, genetic and environmental factors [4]. Unlike other ‘omics’, metabolomic studies directly reflect the underlying biochemical activity and the state of cells/tissues [5,6]. The Human Metabolome Database contains more than 42,000 metabolites, from sugars and peptides to cofactors.

Among the first relevant experimental metabolomic studies on the changes of serum metabolites during systemic inflammatory response, Kamisoglu et al. published his findings in the year 2013 [7]. The metabolomic data set obtained by gas chromatography/mass spectrometry (GC/MS) and liquid chromatography/mass spectrometry (LC/MS) methods included temporal concentration data of 366 plasma metabolites, and 60 of these 366 had significant differential temporal profiles from the Control Group. Recently, several studies have been published on using a metabolomic approach in the diagnosis and prognosis of sepsis [8,9,10]. Kauppi et al. (2016) have published a prospective study where whole blood samples from 65 patients with bacteraemic sepsis and 49 controls were compared [11]. The blood samples were analysed using gas chromatography coupled to time-of-flight (GC/TOF) mass spectrometry. A 6-metabolite predictive logistic regression model showed a sensitivity of 0.91 (95% confidence interval (CI) 0.69–0.99) and a specificity of 0.84 (95% CI 0.58–0.94) with an area under the curve (AUC) of 0.93 (95% CI 0.89–1.01) for bacteraemia. Myristic acid was the single most predictive metabolite, with a sensitivity of 1.00 (95% CI 0.85–1.00) and specificity of 0.95 (95% CI 0.74–0.99) and performed better than various combinations of conventional laboratory and clinical parameters. Cambiaghi et al. (2017) have observed a significant decrease of myristic acid in the plasma of non-responders to the septic shock treatment after 48 h [12]. Servià et al. (2019) observed a significant correlation of increased myristic acid levels and mortality from severe trauma in a prospective metabolomic study including 48 trauma patients [13].

Remarkable findings of the above mentioned studies and our own observation of the kinetics of serum free myristic acid levels following accidental intravenous administration of germfree eubiotic preparation causing a sepsis-like inflammatory response [14] have inspired us to conduct a pilot study into the correlation of free myristic acid serum levels and diagnosis of sepsis with and without bacteraemia, including a comparison with patients with non-infectious SIRS and Control Groups.

To our knowledge, this has been the first study into free myristic acid serum levels determined by the GC/MS method in patients during the first 5 consecutive days following the sepsis or SIRS diagnosis, including a comparison to the free myristic acid serum levels at one-off measurement in a cohort of healthy controls.

Our study has the ambition to turn the attention of intensive care physicians and researchers to this metabolite and to stimulate further studies and research activities in the field.

## 2. Materials and Methods

### 2.1. Study Population

The prospective observational study was conducted from October 2017 to July 2020 at the Department of Anesthesiology and Intensive Care, First Faculty of Medicine, Charles University and Thomayer University Hospital, Prague. The study is registered on ClinicalTrials.gov (NCT03314831). The study was performed with strict adherence to the Helsinki Declaration, and the protocol was approved by the Ethical Committee of the Institute of Clinical and Experimental Medicine and the Thomayer University Hospital Prague, Czech Republic (no. G-17-08-07). Informed consent was obtained from all of the subjects included in the study.

A total number of 196 participants were included in the study. The subjects were divided into three groups according to the predefined criteria—patients with sepsis/septic shock (Sepsis Group); patients with SIRS of non-infectious ethiology (SIRS Group), and controls (Healthy Control Group)-blood donors. The inclusion and exclusion criteria for the Sepsis Group and SIRS Group are presented in Table 1.

#### 2.1.1. Sepsis Group

Patients in the Sepsis Group were included according to the diagnostic criteria for sepsis and septic shock as per the 3rd International Consensus Definitions for Sepsis and Septic Shock (Sepsis-3). Patients who were later found not having sepsis by a retrospective analysis of clinical and laboratory results (wrong initial diagnosis) were excluded from the study. The total of 34 subjects were included in the Sepsis Group. The patients’ characteristics are shown in Table 2.

Patients were treated according to the current guidelines for the treatment of sepsis and septic shock including Surviving Sepsis Campaign guidelines [4].

The antibiotic therapy was started immediately after the sepsis diagnosis was established, i.e., at T0 at the latest. If the offending microorganism was known, a pathogen directed therapy was administered. For unknown microbiological etiology, broad spectrum empirical antibiotics with respect to the probable cause and sensitivity were started, and de-escalated to targeted antimicrobials as soon as the culture results were available.

Patients’ demographic, clinical, and laboratory data were recorded in the usual manner for intensive care unit stay.

Samples for myristic acid level evaluation were collected per protocol at defined time points: On admission (T0), 12 h following admission (T12), on day 1 of ICU stay (D1), on day 3 (D3), and on day 5 (D5).

At T0, D1, D3, and D5, other inflammatory markers including CRP and PCT were measured, as well as other laboratory tests performed according to the standard set of investigations for critically ill patients—arterial blood gas, differential blood count, renal and liver function tests, etc. The sequential organ failure assessment (SOFA) [2] was recorded daily at least until D5, and mortality until D28 was observed.

On admission (T0), blood culture was done in all of the patients, and the focus of infection (known or probable) identified and documented.

#### 2.1.2. SIRS Group

The SIRS Group comprised patients undergoing an elective major surgery fulfilling the SIRS criteria according to the American College of Chest Physicians/Society of Critical Care Medicine Consensus Conference [3] when the infectious origin of SIRS was ruled out. The total of 31 subjects were included in the SIRS Group. The patients’ characteristics are shown in Table 3.

Patients’ demographic, clinical, and laboratory data were recorded in the usual manner for intensive care unit (ICU) stay in the SIRS Group, and in the same pattern as for the patients in the Sepsis Group.

Samples for myristic acid level evaluation were collected per protocol at defined time points: On admission (T0), 12 h following admission (T12), on day 1 of ICU stay (D1), on day 3 (D3), and on day 5 (D5).

At T0, D1, D3, and D5 other inflammatory markers including C-reactive protein (CRP) and procalcitonin (PCT) were measured, as well as other laboratory tests performed according to a standard set of investigations for postoperative patients after major surgery—arterial blood gas, differential blood count, renal and liver function tests, etc.

#### 2.1.3. Healthy Control Group

A total of 120 blood donors were included in the Healthy Subjects Group. All of them met the criteria for blood donation as recommended by the Society for Transfusion Medicine of the Czech Medical Association [5]. The subjects eligible for blood donation but diagnosed with hyperlipidaemia or metabolic syndrome were excluded from the study. The age median (interquartile range) in the Healthy Control Group was 36 (31–41) years. There was 74 (61.7%) of male donors and 46 (38.3%) of female donors in the Healthy Control Group.

### 2.2. Myristic Acid Concentration Analysis

#### 2.2.1. Chemicals

Solvents and reagents including ethyl chloroformate (ECF), pyridine, acetonitril, ethyl alcohol (96%), n-hexan, sodium hydroxide (NaOH), carbonate-bicarbonate solution, sodium sulfate and chloroform were obtained in the best available quality from Fluka AG (Buchs, Switzerland).

#### 2.2.2. Procedure

First, blood samples (Serum sep clot activator Vacuette 5 mL) were collected for myristic acid level evaluation. After centrifugation, aliquots of serum samples were frozen to −35 °C, and later subjected to analysis, which was performed 5–15 days following sampling.

The internal standard solution (2 µL, 10 mmol/L malonic acid in 50 mmol/L HCl) and serum/plasma (0.2 mL) were added to acetonitril (0.4 mL) and ethanol (0.2 mL). The mixture was briefly shaken to precipitate the proteins, and the content was then centrifuged at 2000× *g* for 10 min. The supernatant (0.6 mL approximately) was transferred into another tube, alkalinized to pH > 8 by the addition of 2–3 µL NaOH (2 mol/L) and extracted two times with 0.5 mL n-hexane (shaking for about 1 min) to remove neutral lipids. The hexane layer was aspirated off and 500 µL of the aqueous phase were treated in a silanized glass tube with 20 µL of ECF and 40 µL of pyridine, while shaking the content for a few seconds to let the liberated carbon dioxide escape. Following the addition of 0.25 mL chloroform and 0.5 mL carbonate-bicarbonate solution (1 mol/L, pH ca. 10), the stoppered tube was shaken for about 10 s and left to reach phase equilibrium by standing for 2–3 min. Finally, the upper aqueous layer was aspirated off by means of pipette-tips and the organic phase was dried by adding sodium sulfate (50–80 mg). The volume was reduced to approximately 80–100 µL by blowing nitrogen across the surface of the solvent at room or slightly elevated temperature (40 °C) within 2–3 min. An aliquot of 1–2 µL was then injected onto the fused-silica capillary column 30 m × 0.25 mm of DB-17HT type (0.25 µm film thickness of 50% phenylmethyl silicone; J&W Scientific, Folsom, CA, USA) in the split mode (1:20). The temperature was programmed from 60 °C at 20 °C/min to 300 °C (2 min hold), the flow rate of the helium was 1 mL/min. The spectrometrical analysis was performed by a tandem GC/MS Voyager (Thermo Scientific, Waltham, MA, USA).

### 2.3. Inflammatory Biomarkers and Other Standard Laboratory Tests

Inflammatory biomarker levels including CRP and procalcitonin PCT were measured by electrochemiluminescence (for CRP) and immunoturbidimetry (for PCT). Blood gas analysis, renal and liver function tests, differential blood count were performed according to standard institutional laboratory methods in the participating centers.

### 2.4. Blood Cultures

Blood cultures were collected with a strict observance of aseptic conditions. A single venipuncture, central venous catheter, arterial line or its combination were used to inoculate the aerobic/anaerobic bottle of Bactec Plus (Becton Dickinson, Franklin Lakes, NJ, USA) with 10 mL of whole blood per bottle and processed in the department of clinical microbiology.

At least one pair of bottles (aerobic and anaerobic) was inoculated per patient. Bottles were incubated in the Becton Dickinson BACTEC FX40 (Becton Dickinson, Franklin Lakes, NJ, USA) blood culture system for 7 days. Standard microbial cultivation methods were used to identify the microbial agent. The following criteria were used to assess true bacteraemia: (1) The same pathogen in both blood culture bottles (aerobic and anaerobic) was found; (2) the same microorganism in at least one bottle (aerobic or anaerobic) was found in all sets, and it was not a potential contaminant; (3) the pathogen found in blood culture was corresponding to other culture specimens (sputum, urine, etc.) or (4) the microorganism found in blood culture was corresponding to a confirmed focus of infection outside the bloodstream. If the results missed all of the criteria and the microorganism was potentially contaminant (*coagulase-negative staphylococci*, *Bacillus* spp.,* viridans group streptococci*, *Corynebacterium* spp., *Propionibacterium* spp., *Micrococcus* spp., and *Clostridium perfringens*), the blood culture was considered as a contaminant.

### 2.5. Statistical Analyses

The normality of myristic acid was rejected by the Shapiro-Wilk test, therefore nonparametric tests were applied. The median, minimum, and maximum were used to describe variables. A comparison in time was done by the Wilcoxon test, and Bonferroni correcting was used to adjust significant levels. Between groups, differences were tested by the Kruskal-Wallis test, and Steel-Dwass method was used for pairwise comparisons. To find the optimal values to discriminate between groups, we used the ROC analysis and calculated the AUC value. All of the tests were two-sided, and a p less than 5% was considered as statistically significant. The statistical software JMP^®^ 15.2.0 (SAS Institute Inc., Cary, NC, USA) was used.

## 3. Results

Median values of myristic acid for the respective groups and time points are presented in Table 4.

**Figure 1:**
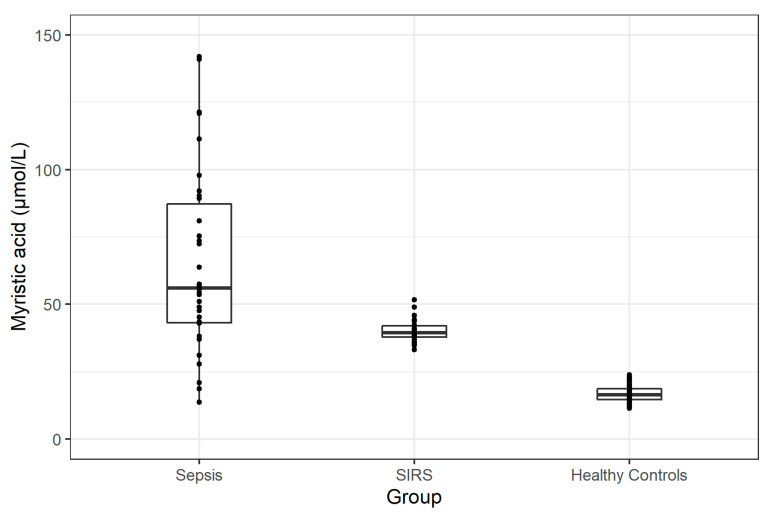
A comparison of peak myristic acid concentrations in the Sepsis, SIRS, and Healthy Control Groups.

The myristic acid levels in the Sepsis and SIRS Groups were found to be significantly higher than in the Healthy Control Group. The significantly higher values were observed both at T0 and in the peak concentrations. Peak concentrations of myristic acid in the Sepsis, SIRS and Control Groups are shown in Figure 1.

Comparing the Sepsis and SIRS Groups, the myristic acid levels only differed in the peak concentrations. No statistical significance was seen in myristic acid levels at T0 (see Table 5).

**Figure 2:**
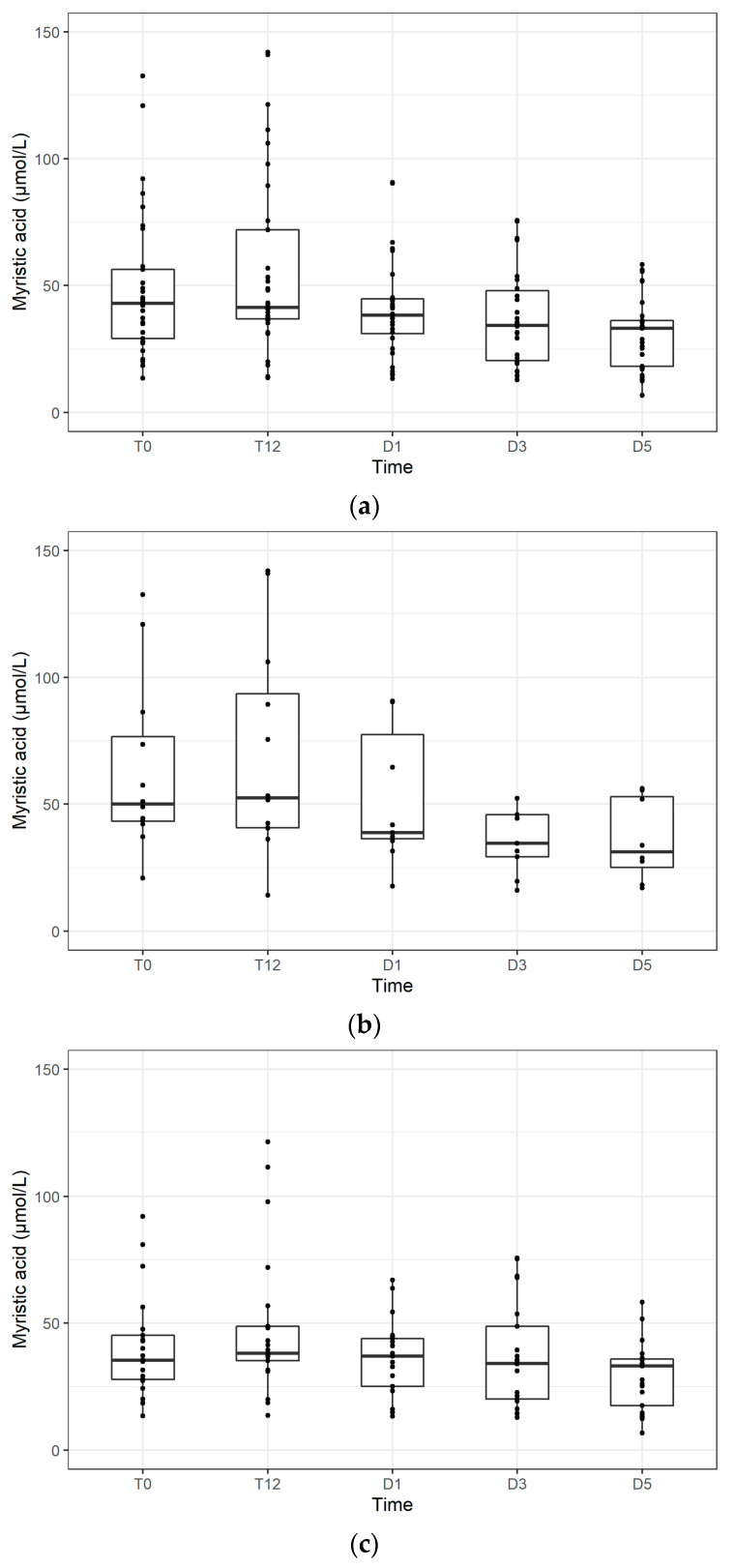
The progress of myristic acid levels at individual times (**a**) in the Sepsis Group as a whole, (**b**) in the subgroup of septic patients with bacteraemia, and (**c**) in the subgroup of septic patients without bacteraemia.

Peak concentrations of myristic acid in the SIRS and Sepsis Groups were reached most frequently at T0, followed by a gradual decline until the fifth day (D5). Most patients in both groups had the highest levels of myristic acid within 24 h after enrollment in the study (T0–T24). Myristic acid levels at individual time points in the Sepsis Group as a whole, and in Sepsis with and without bacteraemia subgroups are shown in Figure 2.

**Figure 3:**
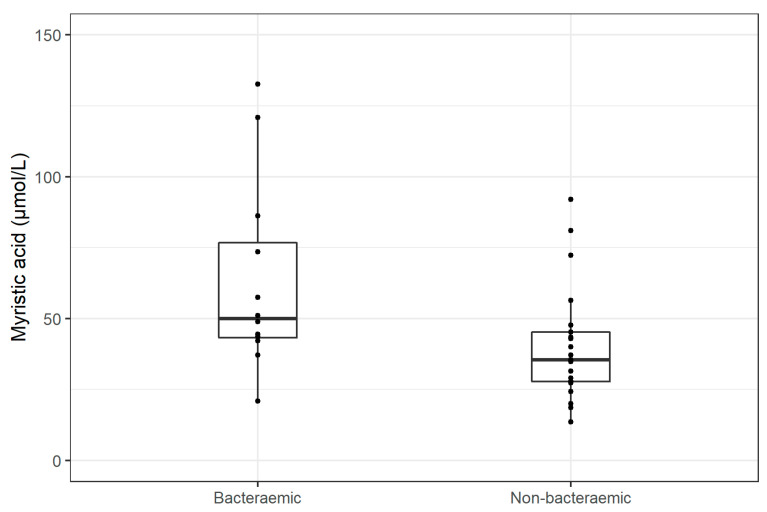
A comparison of myristic acid levels on admission in septic patients with and without bacteraemia.

The serum concentration of myristic acid in septic patients with bacteraemia at T0 was higher than in septic patients without bacteraemia (*p* = 0.016), Figure 3.

**Figure 4:**
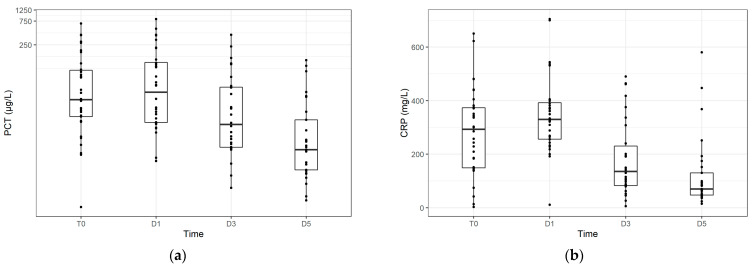
Serum concentrations of procalcitonin (**a**) and C-reactive protein (**b**) at individual times.

**Figure 5:**
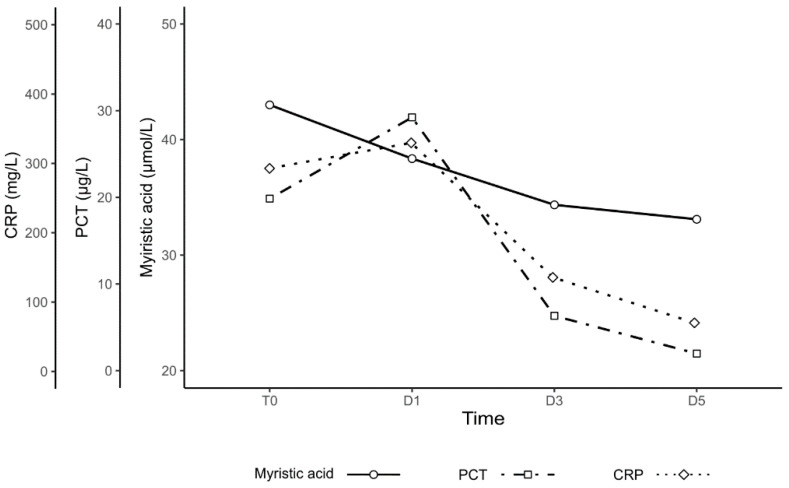
Illustrative timeline of PCT, CRP, and myristic acid levels in the Sepsis Group at T0–D5 samplings. The values are represented as median, the error bars have been left out for lucidity reasons.

CRP and PCT kinetics where typical for sepsis (see Figure 4). Figure 5 compares the CRP, PCT, and myristic acid levels in time.

**Figure 6:**
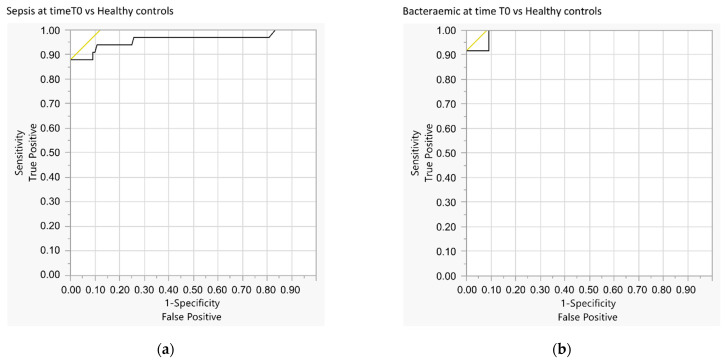

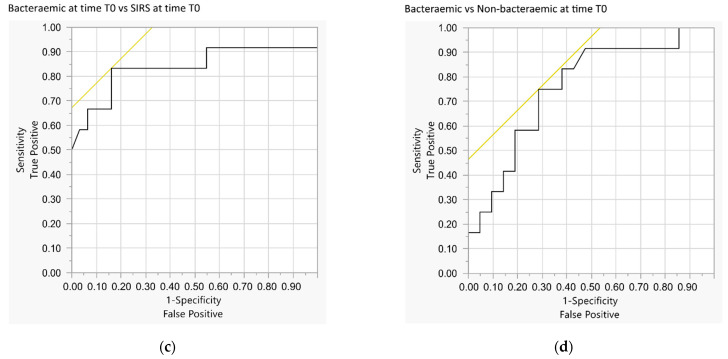
ROC curves for comparisons of selected groups: (**a**) Septic patients on admission vs. healthy controls; (**b**) septic patients with bacteraemia on admission vs. healthy controls; (**c**) septic patients with bacteraemia on admission vs. SIRS patients on admission; and (**d**) septic patients with bacteraemia on admission vs. septic patients without bacteraemia on admission.

Comparing the Sepsis Group patients at T0 with the Healthy Control Group, the ROC analysis identified a myristic acid concentration of 24.3 µmol/L (AUC 0.961) as the cut-off value for prediction of sepsis with 87.9% sensitivity and 100% specificity (see Figure 6a).

By comparison of the septic patients with bacteraemia at T0 with the Healthy Control Group, the ROC analysis determined a myristic acid concentration of 37.2 μmol (AUC 0.992) as the cut-off value for prediction of septic bacteraemia with 91.7% sensitivity and 100% specificity (see Figure 6b).

The cut-off value of 42.1 µmol/L for myristic acid, derived from the ROC analysis (AUC 0.837), discriminated sepsis with bacteraemia from SIRS with 83.3% sensitivity and 83.9% specificity (see Figure 6c).

To predict bacteraemia within the Sepsis Group at T0, the cut-off value of 43.7 µmol/L for myristic acid determined by the ROC analysis (AUC 0.756) showed 75% sensitivity and 71.4% specificity (see Figure 6d).

The cut-off value for myristic acid concentration which discriminates with 100% sensitivity and 100% specificity between SIRS patients and healthy volunteers at T0 lies within interval 23.9–30.5 µmol/L.

Within 28 days of the sepsis diagnosis, six out of 34 patients died (17.6% 28-day mortality) in the Sepsis Group. In the subgroup of sepsis with bacteraemia, four patients out of 12 died (33.3% 28-day mortality). In the subgroup of sepsis without bacteraemia, two patients out of 22 died (9.1% 28-day mortality).

No differences in the myristic acid levels at T0 (*p* = 0.23) and the peak levels (*p* = 0.07) were found between the surviving and dead patients.

## 4. Discussion

The principal findings of our study show that free myristic acid serum levels are significantly increased in patients with systemic inflammatory response and sepsis, confirming the findings of previous metabolomic studies. In accordance with Kauppi et al. (2016), we observed the highest elevation of serum myristic acid levels in septic patients with bacteraemia. The elevation was significantly lower in septic patients without bacteraemia. The myristic acid levels, measured quantitatively, reached their peak values within 24 h following the diagnosis of sepsis and then decreasing until D5 following diagnosis, however, not reaching the values of healthy controls. The ROC analysis determined the cut-off value of 37.2 μmol to predict sepsis with bacteraemia (100% specificity, 92% sensitivity, AUC 0.992), and the cut-off value of 43.7 µmol/L to discriminate between septic patients with and without bacteraemia (75% sensitivity, 71.4% specificity, AUC 0.756). These findings suggest considering to include myristic acid among other inflammatory biomarkers.

The routinely used combination of inflammatory markers PCT and CRP together with clinical signs and tissue perfusion parameters (lactate, pH, base deficit) provide solid information about the clinical course of sepsis. Serum PCT levels reflect the bacterial infection and the severity of the damage it causes. Its accuracy for rapid early detection of bacterial infections in ICU patients is limited by a delay of approximately 24 h between the onset of infection and the rise of PCT. The determination of the optimal cut-off value for the prediction of bacterial infection is cumbersome. In intensive care, the cut-off value of 1.1 ng/mL reaches up to 97% sensitivity and 78% specificity [18]. However, PCT fails to meet the criteria of an ideal biomarker, and also has limitations in its questionable ability to negatively predict bacteremia [19,20,21,22,23]. The timely identification of bacteraemia together with its adequate treatment are crucial for the clinical outcome of septic patients [24]. According to Søgaard et al. (2011), community bacteraemia is connected with a much higher mortality risk within the first 2 days of admission [25]. Laupland et al. (2013) observed the hospital mortality of bacteraemic patients to be up to 60% higher, suggesting that the effect of bacteraemia is mediated to a large extent by the severity of the acute systemic inflammatory response [26].

New molecules studied as potential biomarkers can find clinical application if they provide additional information contributing to the diagnostic-therapeutic approach to the septic patient. From this perspective, the observed increased serum levels of myristic acid in patients with bacteraemia (as previously described in Kauppi’s work) is intriguing and deserves further attention.

In order to consider the use of myristic acid as an inflammatory marker in clinical practice, it is more than desirable to design a timely, technically, and financially reasonable methodology. This study has already used a modified methodology, which brings about shortening of the evaluation procedure, as well as precision of the results [27].

In the 1960s, silylation reagents were introduced into the analytical chemistry and widely accepted as general purpose reagents; their use simplified substantially the derivatization of polyfunctional compounds since the reaction was reduced to the one-step treatment.

In the 1990s, an ability of chloroformates to act as rapid esterification agents of carboxylic acids in the presence of water was discovered and the potentiality of using them as general purpose reagents, too, was tested. Various classes of carboxylic acids were subjected to the treatment with ethyl and methyl chloroformates and the reaction conditions for the formation of esters, carbonates, and carbamates were optimized. Compared to the silylation procedures, there was an additional profit in terms of simplifying the sample workup for the serum/plasma organic acid profiling while getting a more comprehensive information (involving amino acids in the profile). The use of ECF as a derivatizing agent presents a comprehensive approach to profiling organic and amino acids in biological fluids. The sample preparation is made substantially easier, the derivative formation is shortened dramatically (second), and the resulting spectra are considerably clearer and more detailed, thus enabling a much more precise interpretation. Moreover, the serum/plasma can be treated by ECF in situ. The analysis of one sample with the GC/MS ready can be completed within 25 min (including derivatization).

The interesting question remains of the origin and the role of this monosaturated (14:0) organic acid of linear structure with already described atherogenic and thrombogenic potential in sepsis [28,29]. Myristic acid chains are found in different quantities in Lipid A in various species of G negative bacteria [30,31]. The hypothesis that myristic acid is a product of lipopolysaccharide in the degrading cell walls was declined on the grounds of the high myristic levels found even in Gram-positive bacteraemias, as well as due to its significantly raised levels following an accidentally induced sepsis-like systemic reaction to intravenously administered eubiotics, in which myristic acid was not detected [14].

Myristic acid is also a component of cellular membranes. It can be covalently linked to proteins by a process called myristoylation, and serve to anchor signal proteins by insertion of the acyl chain into the lipid bilayer and also to allow the transport of peptides to living cells [32,33,34].

Protein N-myristoylation has shown to be an important evolutionarily conserved modification of proteins involved in various physiological processes, such as cell proliferation, differentiation, survival, and cell death, and can be considered a necessary step in initiating many immune cell signaling cascades [35].

Myristoylated (C 14:0) as well as palmitoylated (C 16:0) proteins are currently widely studied, and it can be assumed that myristic acid, and therefore also palmitic acid, may have important physiological functions [36,37,38,39].

In the light of Fong et al. (1990) [40], it is possible to admit that alterations in lipid metabolism that are related to changes in cellular energy production during inflammatory response activation may be responsible for the elevated levels of myristic acid in the early stages of septic episode [7]. The question still to be answered is, however, ‘why only this fatty acid, and not other shorter or longer chain fatty acid correlate with mortality’, as already pointed out by Servià et al. (2019) [13].

The major limitation of the study is a low number of subjects in the Sepsis and SIRS Groups. The Control Group, on the other hand, is in our view a well-defined homogenous population, and the observed myristic acid levels produce quite robust evidence of normal/physiological concentration.

Heterogenity of the patients in the Sepsis and SIRS Groups, whether in terms of underlying etiology of inflammatory challenge (site of infection, different microbial agents, different cause of SIRS response) or patients‘ population (co-morbid conditions, age) is another potential limitation of the study and its findings.

Kamisoglu et al. (2013) proved changes in the levels of metabolites under observation within the first 24 h following administration of lipopolysaccharide to healthy subjects [7]. Our study was conducted in clinical conditions, therefore the actual onset of sepsis was rarely captured immediately at enrollment into the study, T0, was by various extent delayed. Myristic acid, being a metabolite, presumably reaches peak concentration fairly quickly after the inflammation trigger. For these reasons, our findings concerning myristic acid and other biomarkers concentration levels especially in the timeframe T0-T24 may be rather inconsistent. However, we believe, that despite this methodological flaw due to the clinical nature of the study, we have succeeded to show statistically significant differences in myristic acid concentration in different study populations subgroups within 24 h from enrollment. The only way to precisely evaluate the kinetics and dynamics of myristic acid concentration in early stages of inflammation is the experimental model of inflammation/sepsis in healthy volunteers. In this manner, other septic/inflammatory biomarkers were described and respective findings published by, for instance, Lowry et al. (2005) [41], Calvano et al. (2012) [42], and, as early as in 1994, by Dandona et al., who described the kinetics of serum PCT concentrations following administration of endotoxine to healthy volunteers [43].

Another potential weakness of the study is the sensitivity and specificity of blood culture to detect or rule out bacteraemia. There are many factors contributing to false negative results of blood culture, i.e., sample collection, handling and processing, ongoing antibiotic therapy, and presence of slow growing and/or difficult to culture bacteria. Thus, the results for the subgroup of septic patients without detected bacteraemia may be affected by inclusion of patients with missed bactearemia at T0. In comparison with Kauppi et al. (2016), we have detected more bacterial species in blood in the subgroup of septic patients with bacteraemia [11].

The study limitation is certainly some degree of bias related to the selection of subjects into the Sepsis and SIRS Groups, which was not done in all patients consecutively admitted in the ICU. This would be required to increase the accuracy and objectivity of the study. For continuing research on this topic and validation of our findings prospective randomized studies are needed.

Our pilot clinical study has produced results consistent with the findings of large metabolomic studies, thus contributing to the metabolomic research in sepsis diagnosis and outcome prediction. The well controlled studies in large population cohorts are necessary to gather enough data for a robust and statistically significant conclusion.

In line with the growing expert interest in protein modification by myristoylation, it may be hypothesized, that myristic acid plays a specific physiological part in the mediation of inflammatory response and therefore may become another useful biomarker of SIRS [32,35,37,38,44,45]. We have observed a significant increase in the myristic acid level in patients with systemic inflammation of non-infectious etiology clearly discriminating (100% sensitivity and specificity) this cohort from healthy controls.

Our group continues the research in myristic acid levels correlation with infection and inflammation, extending the study population by inclusion of patients with the systemic inflammatory response of non-infectious etiology and receiving an immunosuppressive treatment after transplantation of solid organs. It is well known and confirmed by our own observations, that the levels and kinetics of inflammatory markers in these patients are modified [21,46,47].

Our study provides yet another piece of evidence, that metabolomic studies may contribute to the clinical management of patients with severe inflammation. The metabolomic research has all the prerequisites to produce new time-sensitive and specific biomarkers useful for sepsis/SIRS diagnosis and outcome prediction.

## Figures and Tables

**Table 1:** Inclusion and exclusion criteria for the Sepsis and SIRS Groups.

Group	Inclusion Criteria	Exclusion Criteria
Sepsis	At least two qSOFA criteria present [15]: - respiratory rate ≥ 22/min - mental state alteration - systolic blood pressure ≤ 100 mm Hg At least two point rise in SOFA [16] Infection clinically suspected	Moribund patient expected to die within 24 h After cardiopulmonary resuscitation or prolonged severe hypoperfusion Immunodeficiency (hematologic malignancies, HIV, congenital) Age < 18 years
SIRS	­ Patients undergoing elective surgery At least two criteria present [17]: - temperature > 38 °C or < 36 °C - pulse rate > 90/min - respiratory rate > 20/min or PaCO_2_ < 4.3 kPa - leucocytes > 12,000/mL or < 4000/mL or > 10% immature forms	Immunosuppression Infection proved within 10 days following surgery Death within 30 days following surgery Age < 18 years

qSOFA—quick Sepsis Related Organ Failure Assessment; SOFA—Sequential Organ Failure Assessment.

**Table 2:** Study population characteristics for the Sepsis Group.

Age (years)	65 (48–69) *
Male gender (*n*, %)	23 (67.6)
Female gender (*n*, %)	11 (32.4)
Bacteraemia (*n*, %)	12 (35.3)
Without bacteraemia (*n*, %)	22 (64.7)
Comorbid conditions (*n*)	
arterial hypertension	28
asthma bronchiale	6
chronic kidney disease	7
chronic obstructive pulmonary disease	2
hyperlipidaemia	12
hypothyroidism	6
ischemic heart disease	7
type 1 diabetes	1
type 2 diabetes	16
Infectious focus (*n*, %)	
gastrointestinal	9 (26.5)
soft tissue/bone	2 (5.9)
respiratory	14 (41.2)
urinary tract	5 (14.7)
mixed	4 (11.8)
Blood culture results (*n*, %)	
Clostridium sp.	1 (6.3)
Enterobacter cloacae	1 (6.3)
Escherichia coli	4 (25)
Klebsiella pneumoniae	3 (18.8)
Proteus mirabilis	1 (6.3)
Staphylococcus aureus	1 (6.3)
Staphylococcus epidermidis	1 (6.3)
Streptococcus pneumoniae	2 (12.5)
Streptococcus pyogenes	2 (12.5)
Mechanical ventilation (*n*, %)	30 (88.2)
Septic shock (*n*, %)	24 (70.6)
SOFA at T0 (points)	9 (8–12)

* Values are displayed as median (interquartile range).

**Table 3:** Study population characteristics for the SIRS Group.

Age (years)	66 (61–72) *
Male gender (*n*, %)	19 (61.3)
Female gender (*n*, %)	12 (38.7)
SIRS without malignancy (*n*, %)	16 (51.6)
SIRS with malignancy (*n*, %)	15 (48.4)
Type of surgery (*n*, %)	
A. illiaca communis aneurysm	1 (3.2)
Abdominal aorta aneurysm	6 (19.4)
Lower limb ischemia	2 (6.5)
Polycystic kidney disease	1 (3.2)
Ileus	1 (3.2)
Vena portae trombosis	1 (3.2)
Chronic pancreatits	1 (3.2)
Ileostomy	1 (3.2)
Short bowel syndrome	1 (3.2)
A. illiaca communis thrombosis	1 (3.2)
Colorectal carcinoma	5 (16.1)
Liver tumour	5 (16.1)
Kidney tumour	1 (3.2)
Pancreatic tumour	3 (9.7)
Small bowl Gastrointestinal stromal tumor	1 (3.2)

* Values are displayed as median (interquartile range).

**Table 4:** Serum levels of myristic acid (µmol/L) for individual groups and times. Values are displayed as medians (interquartile range).

(Sub-)Group	Serum Concentration of Myristic Acid (µmol/L)					
	T0	T12	D1	D3	D5	Peak
Sepsis Group in Total	43.0 (28.6–57.0)	41.3 (36.9–72.0)	38.4 (31.0–44.8)	34.4 (20.5–48.0)	33.1 (18.2–36.3)	56.0 (41.8–89.5)
Sepsis with Bacteraemia	50.0 (42.5–83.0)	52.5 (40.7–93.5)	38.8 (36.4–77.4)	34.6 (29.3–45.8)	31.3 (25.2–52.9)	65.5 (49.5–113.2)
Sepsis without Bacteraemia	35.4 (27.6–46.5)	38.2 (35.2–48.8)	37.1 (25.1–43.9)	34.1 (20.2–48.8)	33.1 (17.5–35.9)	54.0 (37.9–76.7)
SIRS	37.7 (35.6–40.3)		37.3 (34.7–38.7)	36.2 (34.1–37.3)	36.4 (34.6–37.7)	39.5 (37.7–42.3)
Healthy Subjects	16.5 (14.6–18.7)					

**Table 5:** Statistical significance of differences in myristic acid levels between the Sepsis, SIRS, and Healthy Control Groups.

Groups	T0	Peak
Sepsis—Healthy Subjects	*p* < 0.0001	*p* < 0.0001
Sepsis—SIRS	*p* = 0.5	*p* = 0.0006
SIRS—Healthy Subjects	*p* < 0.0001	*p* < 0.0001

## Data Availability

Publicly available datasets were analyzed in this study. This data can be found here: http://arkftn.cz/research/001/Zazula_Myristic_acid_serum_levels-Data.xlsx (accessed on 8 April 2021).
